# Clinicopathologic Features and Cytologic Correlation of *ALK*-Rearranged Papillary Thyroid Carcinoma: A Series of Eight Cases

**DOI:** 10.1007/s12022-024-09808-1

**Published:** 2024-04-20

**Authors:** Kun-Ping Shih, Yu-Cheng Lee, Jia-Jiun Tsai, Shu-Hui Lin, Chih-Yi Liu, Wan-Shan Li, Chien-Feng Li, Jen-Fan Hang

**Affiliations:** 1 Department of Pathology and Laboratory Medicine, Taipei Veterans General Hospital, Shipai Rd, No. 201, Sec. 2, Taipei, 11217 Taiwan; 2 Department of Pathology, National Taiwan University Hospital Hsinchu Branch, Hsinchu, Taiwan; 3 Department of Pathology, Changhua Christian Hospital, Changhua, Taiwan; 4 Department of Post-Baccalaureate Medicine, College of Medicine, National Chung Hsing University, Taichung, Taiwan; 5 Division of Pathology, Sijhih Cathay General Hospital, New Taipei City, Taiwan; 6 School of Medicine, College of Medicine, Fu Jen Catholic University, New Taipei City, Taiwan; 7 Department of Pathology, Chi Mei Medical Center, Tainan, Taiwan; 8 Department of Medical Technology, Chung Hwa University of Medical Technology, Tainan, Taiwan; 9 Department of Medical Research, Chi Mei Medical Center, Tainan, Taiwan; 10 National Institute of Cancer Research, National Health Research Institutes, Tainan, Taiwan; 11 School of Medicine, National Yang Ming Chiao Tung University, Taipei, Taiwan; 12 Institute of Clinical Medicine, National Yang Ming Chiao Tung University, Taipei, Taiwan

**Keywords:** *Anaplastic lymphoma kinase*, Papillary thyroid carcinoma, Fine needle aspiration, The Bethesda System

## Abstract

*Anaplastic lymphoma kinase* (*ALK*) gene fusions are rare in papillary thyroid carcinoma (PTC) but may serve as a therapeutic target. This study aims to evaluate the preoperative cytologic findings and clinicopathologic features of a series of eight *ALK*-rearranged PTCs from our pathology archives and consultations. All cases were confirmed by ALK D5F3 immunohistochemistry and six with additional targeted RNA-based next-generation sequencing (NGS). The original fine-needle aspiration (FNA) cytology diagnosis included the Bethesda System (TBS) category II in three (37.5%), TBS III in two (25%), TBS V in two (25%), and TBS VI in one (12.5%). Six cases had available FNA cytology and were reviewed. The cytologic features showed microfollicular architecture as well as limited or reduced nuclear elongation and chromatin alterations in all six. Nuclear grooves and pseudoinclusions were absent in two cases, rarely or focally noted in three, and frequently found in one. Two cases initially diagnosed as TBS II, showing microfollicular architecture without well-developed nuclear features, were revised to TBS III (with architectural atypia only). For histologic correlations, four were infiltrative follicular variant PTCs, three as classic subtype PTC with predominant follicular growth, and one as solid/trabecular subtype PTC. All eight cases demonstrated reduced PTC nuclear features with respect to nuclear elongation and chromatin alterations compared to those typically identified in “*BRAF*-like” PTCs. The NGS testing revealed *EML4::ALK* fusion in three, *STRN::ALK* fusion in two, and *ITSN2::ALK* fusion in one. In conclusion, although *ALK*-rearranged PTCs have been associated with neutral gene expression profile from a *BRAF-RAS* scoring perspective, the “*RAS*-like” nuclear features were more commonly identified in this series, resulting in frequent indeterminate diagnosis of preoperative FNA.

## Introduction

Papillary thyroid carcinoma (PTC) is the most common malignancy of the thyroid [1]. The majority of PTC harbors *BRAF* mutations, reported in 59.7 to 86.8% [2–4]. In addition, kinase gene fusions are also commonly detected in about 8.5 to 15.3% of PTCs [2–4]. Among them, the *RET* fusions are the most frequent, followed by *NTRK* and *BRAF* [4]. *Anaplastic lymphoma kinase* (*ALK*) belongs to the gene family of receptor tyrosine kinases, which control various cell functions, such as cell proliferation and survival. *ALK* gene rearrangement has been reported in many tumors across various organs, including anaplastic large cell lymphoma, inflammatory myofibroblastic tumor, and non-small cell lung carcinoma [5, 6]. PTCs with *ALK* fusion have been reported in 0–2.2% of unselected patients, 2–7.3% in pediatric and young adult patients, and up to 12.7% in patients with radiation exposure [7–11]. Although PTC generally has a favorable prognosis, distant metastasis and disease progression can occur in a subset of patients. Due to the rarity of *ALK*-rearranged PTCs, their clinical behaviors were still not well-characterized. In addition, *ALK* fusion can serve as a potential therapeutic target given the targeted tyrosine kinase inhibitors. Therefore, identifying *ALK* fusion in PTCs has important clinical implications in the era of precision medicine.

The *ALK*-rearranged PTCs were first discovered in atomic bomb survivors in 2012 and these tumors frequently showed a peculiar solid/trabecular-like architecture [11]. Since then, various histologic subtypes have been described in *ALK*-rearranged PTCs, including follicular variant as well as classic, solid/trabecular, diffuse sclerosing, tall cell, oncocytic, and Warthin-like subtypes [8, 11]. In addition, relatively subtle nuclear atypia has also been observed [8, 12]. In fine-needle aspiration (FNA) cytology, subtle cytologic atypia with mild nuclear enlargement and less frequent nuclear grooves were noted [13]. To the best of our knowledge, the description of cytologic and histologic findings of *ALK*-rearranged PTCs was mostly mentioned in separate studies. The detailed cytomorphologic features as well as cytology-histology correlations were not well reported. In this study, we investigated the cytologic features, the distribution of the Bethesda System (TBS) categories, and the histopathological characteristics of these tumors in a series of eight *ALK*-rearranged PTCs.

## Material and Methods

### Patient Selection

This study was approved by the Institutional Review Board (IRB) of Taipei Veterans General Hospital (IRB no.: 2019-07-001BC) and Changhua Christian Hospital (IRB no.: 230902). This series consisted of cases identified from our previous studies of *BRAF* p.V600E wild-type PTCs, routine cases, and consultations [4, 14]. The *BRAF* p.V600E status was evaluated by a validated immunohistochemical assay (clone VE1, Spring Bioscience, Pleasanton, CA, USA). Previous study has showed ALK immunohistochemistry (IHC) with good sensitivity and specificity for detecting *ALK*-rearranged PTCs [8]. Thus, ALK IHC (clone D5F3, Ventana, Oro Valley, AZ) was applied as the primary diagnostic tool on *BRAF* p.V600E wild-type PTCs in this series. The ALK IHC-positive cases passed the initial quality check for RNA were subjected to targeted RNA next-generation sequencing (NGS) using FusionPlex pan solid tumor v2 (ArcherDX, Boulder, CO) to identify the specific fusion partner genes. Clinical characteristics, including surgical procedure, age, sex, tumor size, TNM stage, dose of radioactive iodine therapy, and follow-up status, were recorded from the medical charts.

### Cytology and Pathology Examination

The preoperative FNA specimens were prepared either by conventional or SurePath (Becton Dickinson, Franklin Lakes, NJ) methods, and were stained with Papanicolaou and/or Liu stains. The diagnosis was based on the 2023 Bethesda System for Reporting Thyroid Cytopathology, including TBS I: nondiagnostic, TBS II: benign, TBS III: atypia of undetermined significance (AUS), TBS IV: follicular neoplasm, TBS V: suspicious for malignancy, and TBS VI: malignant. The TBS III category was further divided into AUS-nuclear atypia and AUS-other [15]. All available FNA slides were reviewed. Architecture features (macrofolliclular, microfollicular, and papillary patterns), nuclear features (elongation, chromatin alterations (powdering/margination), groove, and pseudoinclusion), and the amount of colloid were examined.

All available pathologic slides were reviewed. The diagnosis of PTC and histological subtypes was based on the 2022 World Health Organization classification. Histological features including proportions of growth patterns (macrofollicular, microfollicular, papillary, and solid/trabecular), nuclear features (elongation, chromatin alterations (powdering/margination), groove, and pseudoinclusion), tumor border, the presence of psammoma body, extrathyroidal extension, lymphatic invasion, vascular invasion, and background thyroid parenchyma were recorded. Tumor border was classified into three types: well-circumscribed, multinodular permeative (invasive growth in a multinodular pattern), and infiltrative (irregular and spiculated tumor invasion with desmoplastic change).

## Results

### Clinical Information

**Fig. 1:**
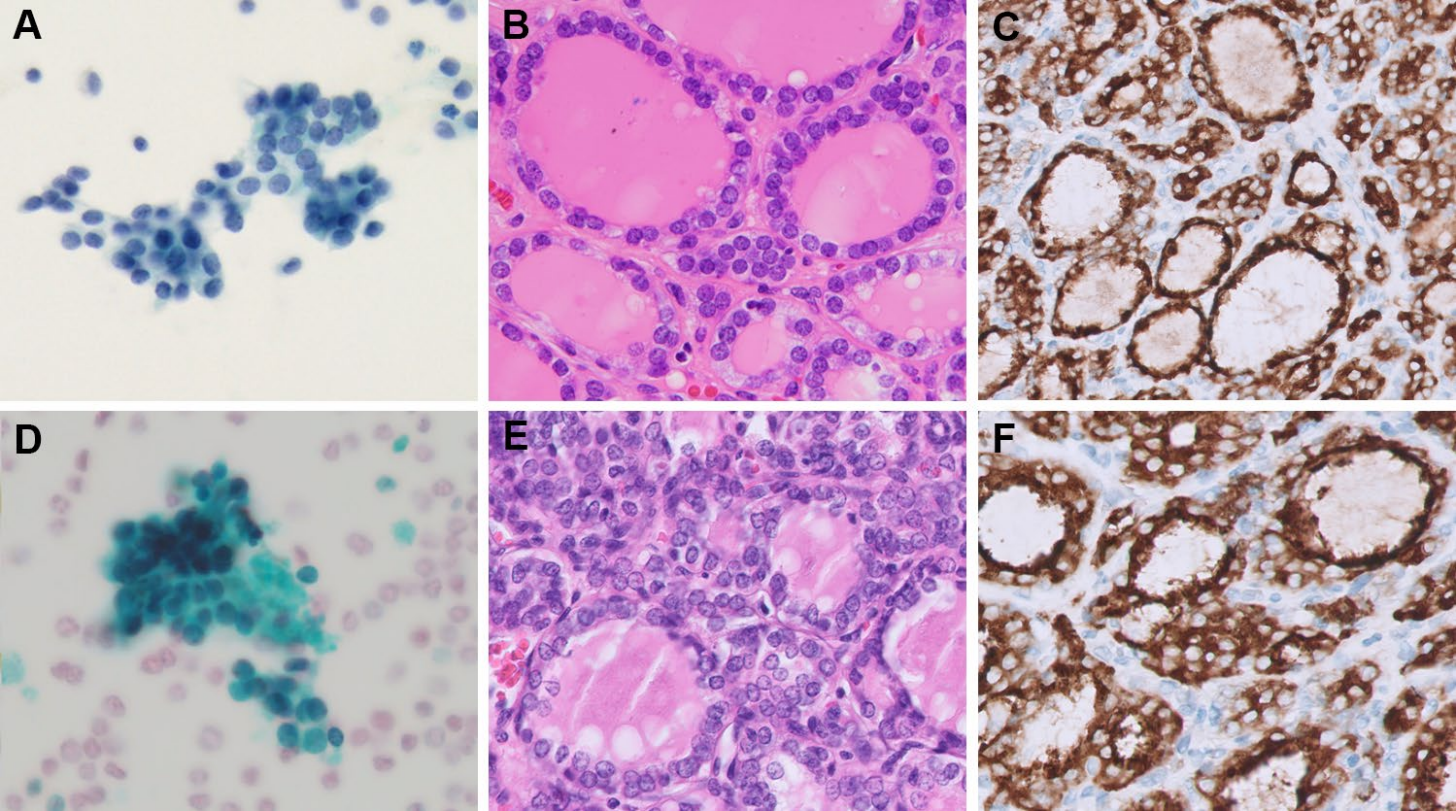
*ALK*-rearranged PTCs originally assigned as TBS II in FNA cytology were reclassified as TBS III: AUS-other (with architectural atypia only) after review. Case 2 showed some microfollicles with “*RAS*-like” PTC nuclear alterations in FNA cytology (**A**, Papanicolaou stain, 400 ×). The corresponding histology showed macrofollicular and microfollicular growth, limited nuclear elongation and chromatin alterations (**B**, H&E, 400 ×), and diffuse and strong cytoplasmic expression of ALK IHC (**C**, ALK, 400 ×). Case 6 showed focal microfollicles and subtle “*RAS*-like” PTC nuclear alterations in FNA cytology (**D**, Papanicolaou stain, 400 ×). The corresponding histology showed predominant microfollicular growth and attenuated nuclear alterations compared to well-developed “*BRAF*-like” nuclear alterations (**E**, H&E, 400 ×) as well as diffuse and strong cytoplasmic expression of ALK IHC (**F**, ALK, 400 ×)

**Fig. 2:**
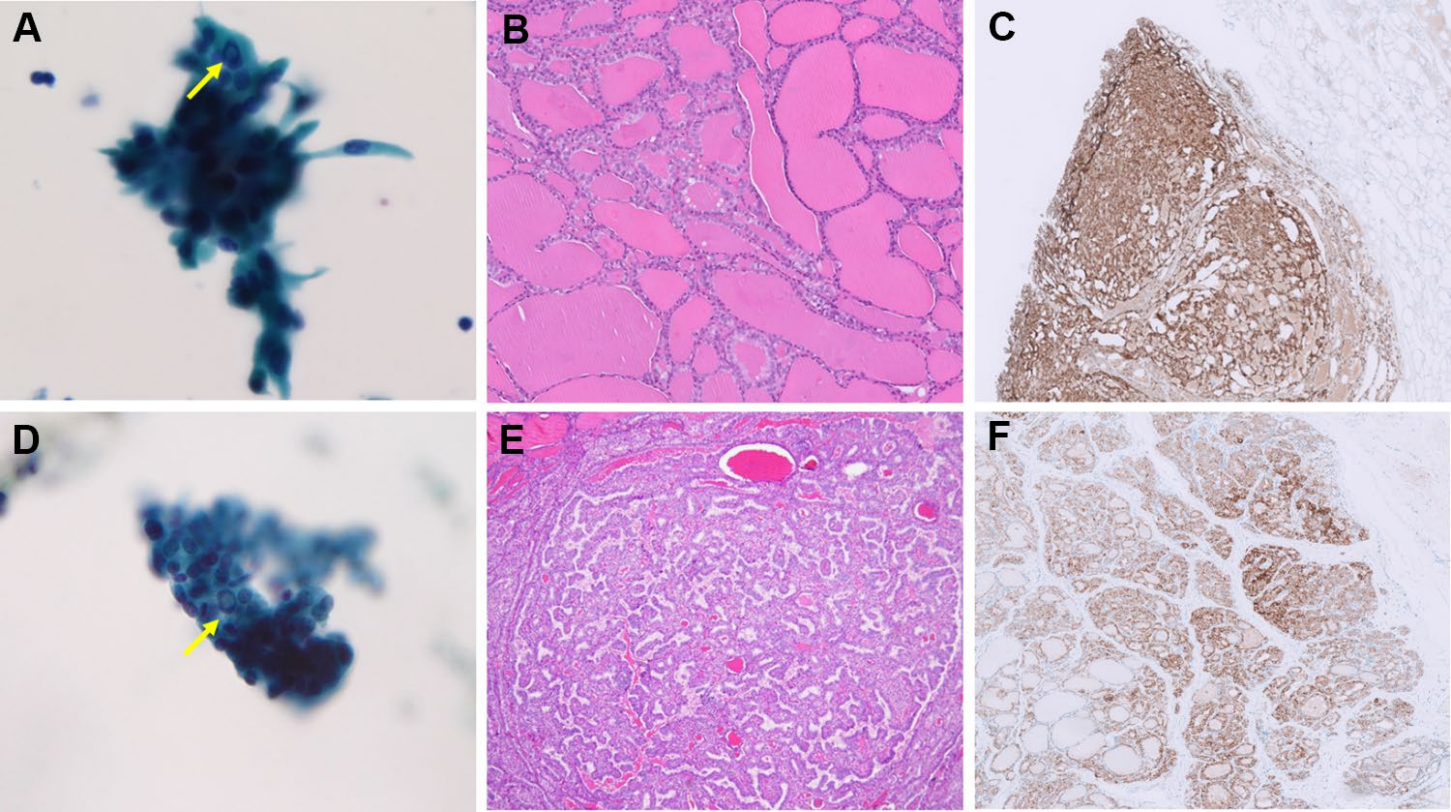
*ALK*-rearranged PTCs classified as TBS V in FNA cytology. Case 5 showed focal nuclear pseudoinclusion (arrow) in FNA cytology (**A**, Papanicolaou stain, 400 ×), predominant macrofollicular growth in histology (**B**, H&E, 200 ×), and diffuse and strong cytoplasmic expression of ALK IHC (**C**, ALK, 40 ×). Case 4 showed focal nuclear pseudoinclusion (arrow) in FNA cytology (**D**, Papanicolaou stain, 400 ×), focal papillary growth in histology (**E**, H&E, 40 ×), and moderate to strong cytoplasmic expression of ALK IHC (**F**, ALK, 40 ×)

Eight *ALK*-rearranged PTCs were identified in this study. Cases 1 to 4 were identified through ALK IHC screening on the tissue microarrays of 60 *BRAF* p.V600E wild-type PTCs from our previous study cohort [14]. Case 5 was diagnosed as *ALK*-rearranged PTC in our routine practice and had been reported in our prior study [4]. Cases 6 to 8 were from the corresponding author’s consultation files. The clinicopathologic features are summarized in Table 1. None of the eight cases had a history of radiation exposure. Six patients underwent total thyroidectomy and two received lobectomy. The average age was 39 years (range: 17–59 years). The female-to-male ratio was 6:2. The average tumor size was 2.2 cm (range: 0.9–4.5 cm). The pT stage included pT1a in three, pT1b in two, pT2 in one, pT3a in one, and pT4a in one. The pT4a case (case 3) showed tracheal invasion and lost to follow-up after the surgery. Six patients were subjected to lymph node dissection. Central compartment cervical lymph node metastasis (N1a) was found in two cases (cases 7 and 8) and lateral compartment cervical lymph node metastasis (N1b) was found in one case (case 2). Six patients received post-operation radioactive iodine therapy. Seven of the eight cases had no sign of disease recurrence and remained free of disease upon follow-up with a mean interval of 47.4 months (4–87 months). The ALK IHC showed diffuse moderate to strong cytoplasmic expression in all eight cases (Figs. 1C and F, 2C and F). Targeted RNA NGS was successfully performed in six cases and the fusion variants included *EML4::ALK* in three, *STRN::ALK* in two, and *ITSN2::ALK* in one. The remaining two cases failed the test due to poor RNA quality.

**Table 1:** The clinicopathologic features of *ALK*-rearranged papillary thyroid carcinoma (PTC)

Case No.	Surgical procedure	Age	Sex	Final pathologic diagnosis	Tumor size (cm)	T	N	RAI therapy	Follow-up status	ALK IHC	Targeted RNA NGS
1	TT	59	M	STPTC	0.9	1a	0	100 mCi	NED (86 months)	Positive	*ITSN2::ALK*
2	TT	17	F	IFVPTC	2.0	1b	1b	80 mCi	NED (75 months)	Positive	*EML4::ALK*
3	TT	49	M	IFVPTC	3.5	4a	0	120 mCi	Lost of follow-up	Positive	*STRN::ALK*
4	TT	55	F	CPTC with predominant follicular growth	3.6	2	0	100 mCi	NED (87 months)	Positive	Failed
5	UL	31	F	CPTC with predominant follicular growth	1.4	1b	n/a	No	NED (18 months)	Positive	*EML4::ALK*
6	UL	20	F	CPTC with predominant follicular growth	4.5	3a	n/a	No	NED (15 months)	Positive	*EML4::ALK*
7	TT	35	F	IFVPTC	1	1a	1a	100 mCi	NED (4 months)	Positive	Failed
8	TT	46	F	IFVPTC	1	1a	1a	120 mCi	NED (47 months)	Positive	*STRN::ALK*

*RAI* radioactive iodine, *IHC* immunohistochemistry, *NGS* next-generation sequencing, *TT* total thyroidectomy, *STPTC* solid/trabecular subtype PTC, *IFVPTC* infiltrative follicular variant PTC, *CPTC* classic subtype PTC, *UL* unilateral lobectomy, *NED* no evidence of disease

### Cytological Features

**Fig. 3:**
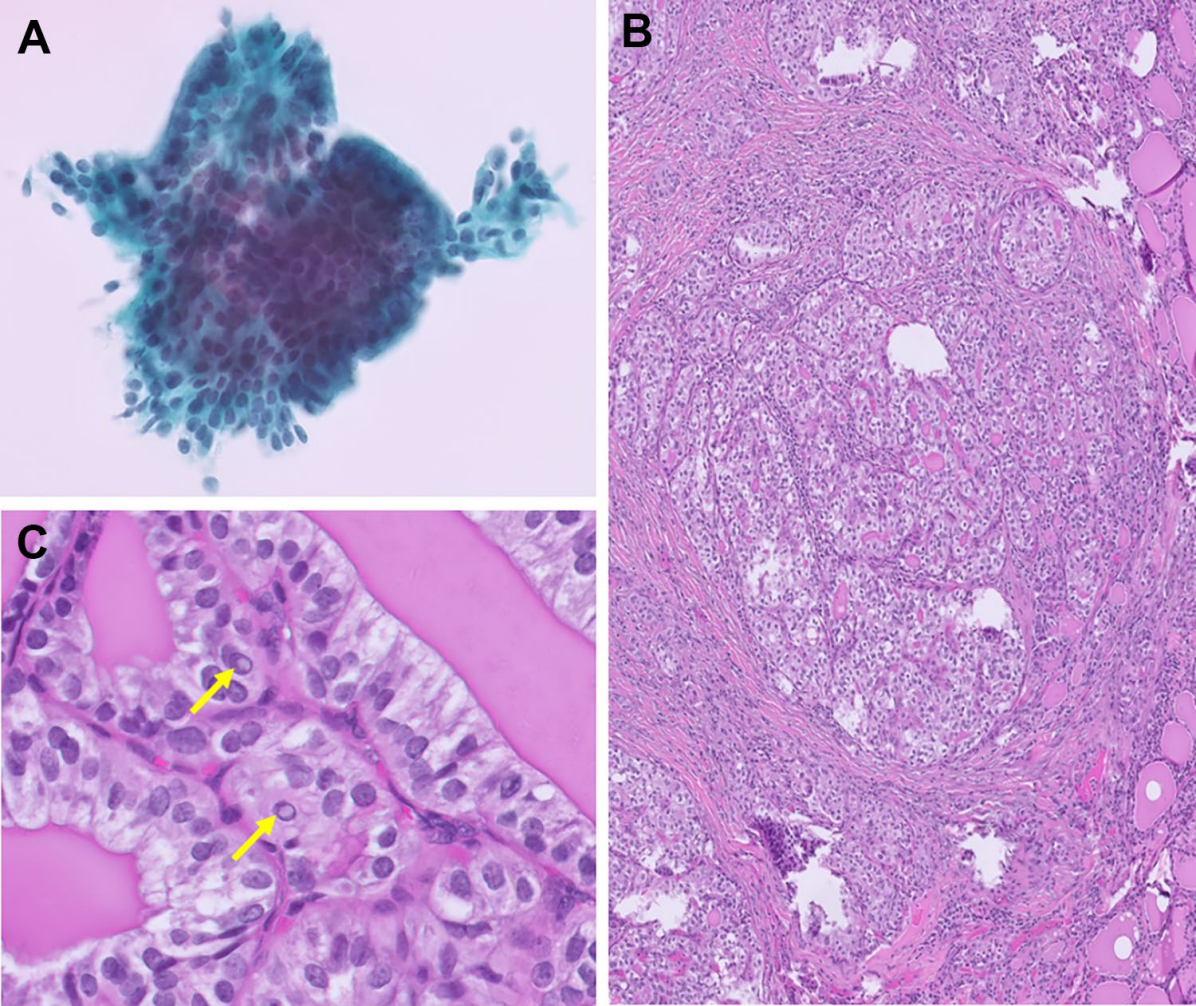
*ALK*-rearranged PTC classified as TBS III in FNA cytology (case 8). The FNA cytology revealed papillary and microfollicular architecture and some nuclear elongation (**A**, Papanicolaou stain, 400 ×). The tumor showed microfollicular with foci of solid/trabecular growth (**B**, H&E, 40 ×). The tumor cells showed reduced chromatin alterations but frequent pseudoinclusions (arrows) and nuclear grooves (**C**, H&E, 400 ×)

**Fig. 4:**
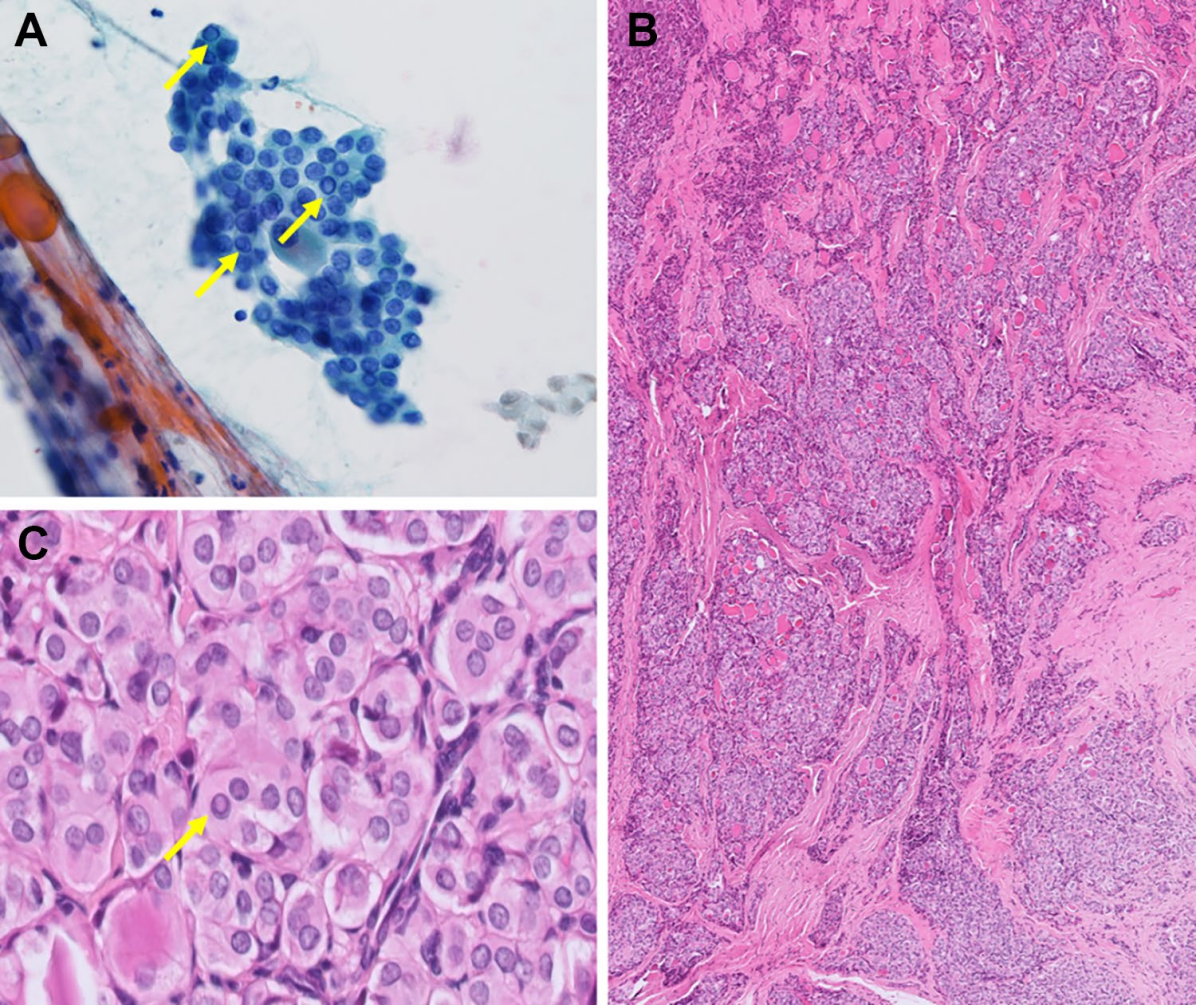
*ALK*-rearranged PTC classified as TBS VI in FNA cytology (case 1). The FNA cytology revealed frequent nuclear pseudoinclusions (arrows) and grooves (**A**, Papanicolaou stain, 400 ×). The tumor showed multinodular permeative tumor border and microfollicular with predominant solid/trabecular growth (**B**, H&E, 40 ×). The tumor cells showed reduced nuclear elongation and chromatin alterations but frequent nuclear pseudoinclusions (arrow) and grooves (**C**, H&E, 400 ×)

The cytologic features of *ALK*-rearranged PTCs are summarized in Table 2. Preoperative FNA was performed in all eight cases and the original diagnosis included TBS II in three, TBS III in two, TBS V in two, and TBS VI in one. The FNA of case 3 (with TBS III diagnosis) was not performed in our country and the remaining seven cases had available FNA slides for review. The FNA from case 7 (with TBS II diagnosis) showed bland follicular cells without PTC nuclear features in a lymphocytic background and very scant colloid. Thus, the findings suggested lymphocytic thyroiditis rather than PTC. It is assumed that the tumor cells were not adequately represented through the FNA and thus the case was removed for the following analysis. In the two cases (cases 2 and 6) (Fig. 1A and D) assigned initially as TBS II, the nuclear features were limited but focal microfollicular architecture was identified. Thus, the diagnosis was revised to TBS III (AUS-other, with architecture atypia only). In case 8 assigned initially as TBS III, the cytology showed focal papillary architecture and rare nuclear atypia (Fig. 3A). Therefore, we assigned the diagnosis as TBS III (AUS-nuclear atypia) after review. The final reviewed diagnosis of the six cases showed TBS III: AUS-nuclear atypia in 16.7% (1/6), TBS III: AUS-other in 33% (2/6), TBS V in 33.3% (2/6), and TBS VI in 16.7% (1/6). Microfollicular architecture was identified in all six cases. Scant papillary architecture and macrofollicular architecture were present in only one case each (case 8 and case 2, respectively). The typical PTC nuclear features of grooves and pseudoinclusions were absent in two cases (Fig. 1A and D), rarely seen in one case, focally found in two cases (Fig. 2A and D), and readily identified in one case (Fig. 4A). Nuclear elongation and chromatin alterations were limited in two cases (Fig. 1A and D) and reduced in the remaining four cases (Figs. 2A and D, 3A, and 4A).

**Table 2:** The cytologic features of *ALK*-rearranged papillary thyroid carcinoma

Case No.	Original TBS	Reviewed TBS	Architecture	Nuclear features				Colloid
				Elongation	Chromatin alterations	Groove	Pseudoinclusion	
1	VI	VI	Microfollicular	Reduced	Reduced	Frequent	Frequent	Scant, dense
2	II	III (AUS-O)	Macrofollicular and microfollicular	Limited	Limited	Absent	Absent	Scant
3	III	n/a	n/a	n/a	n/a	n/a	n/a	n/a
4	V	V	Microfollicular	Reduced	Reduced	Focal	Focal	Scant, dense
5	V	V	Microfollicular	Reduced	Reduced	Focal	Focal	Scant
6	II	III (AUS-O)	Microfollicular	Limited	Limited	Absent	Absent	Scant
7	II	II	n/a*	n/a*	n/a*	n/a*	n/a*	n/a*
8	III	III (AUS-N)	Microfollicular and papillary	Reduced	Reduced	Rare	Rare	Scant

*TBS* the Bethesda System, *AUS-O* atypia of undetermined significance associated with other patterns, *AUS-N* atypia of undetermined significance with nuclear atypia

^*^Not evaluated due to missed sampling of the tumor

### Pathological Features

**Fig. 5:**
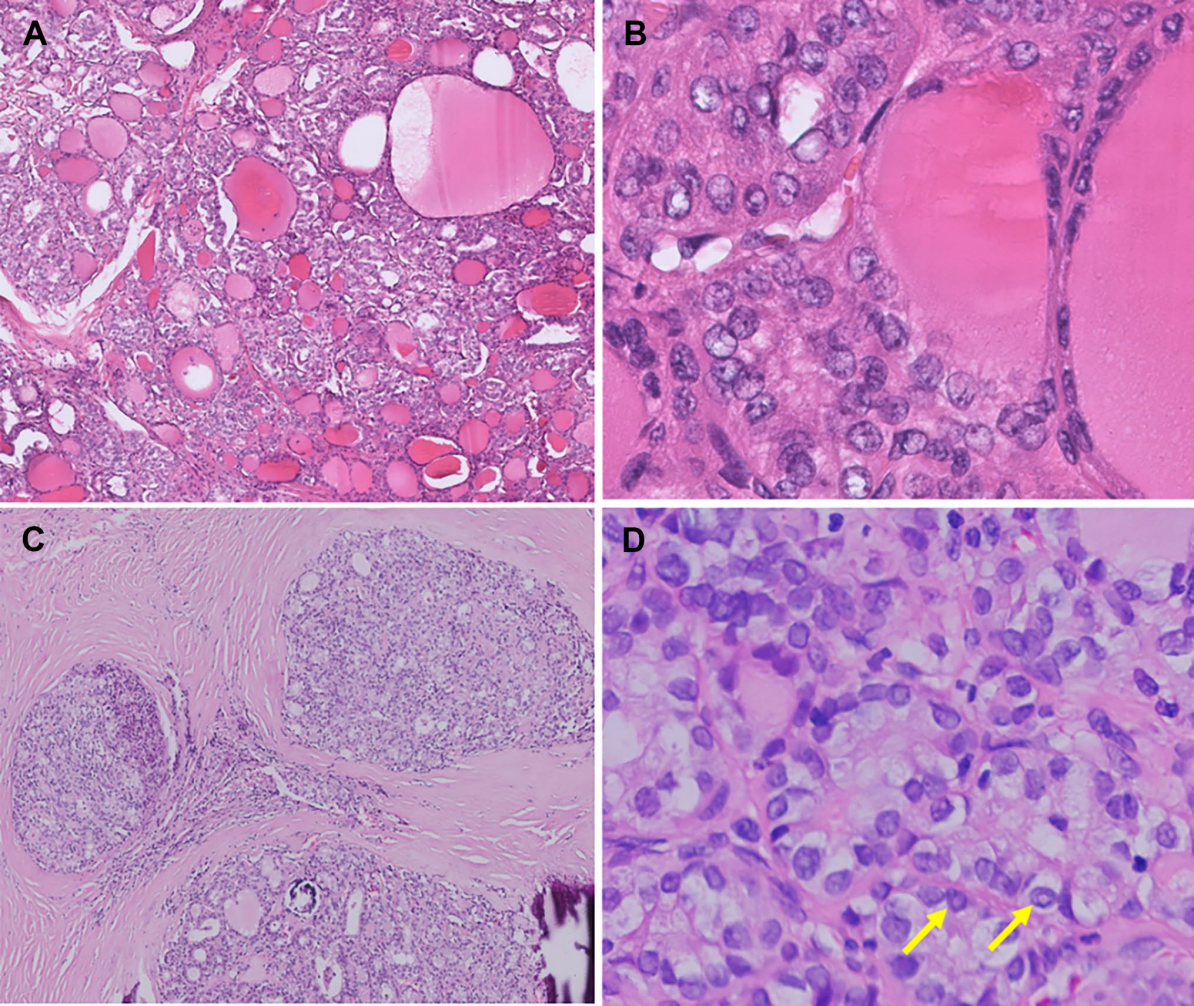
Case 3 showed macrofollicular and microfollicular growth (**A**, H&E, 40 ×). The tumor cells had reduced chromatin alterations and focal nuclear grooves (**B**, H&E, 400 ×). Case 7 had microfollicular growth (**C**, H&E, 40 ×). The tumor cells showed reduced chromatin alterations and irregularities of the nuclear contour but with frequent nuclear pseudoinclusions (arrows) and grooves (**D**, H&E, 400 ×)

The pathologic features of *ALK*-rearranged PTCs are summarized in Table 3. All eight cases demonstrated a predominant follicular growth. Four cases (case 2, 3, 7, and 8) showed a multinodular permeative tumor border and were classified as infiltrative follicular variant PTC (Figs. 3B and 5C). One case (case 1) showed microfollicular with predominant solid/trabecular growth and was diagnosed as solid/trabecular subtype PTC (Fig. 4B). The three remaining cases (case 4, 5, and 6) showed discernible papillary architecture (Fig. 2E) that precludes infiltrative follicular variant PTC and non-invasive follicular thyroid neoplasm with papillary-like nuclear features. Thus, these three cases were diagnosed as classic subtype PTC with predominant follicular growth. Case 5 was the only one that showed well-demarcated tumor border (Fig. 2C), while the other two cases had infiltrative border. Macrofollicular (Figs. 2B and 5A) and microfollicular (Figs. 1B and E, 3B, 4B, and 5C) growths comprised varies proportion of < 5 to 80% and 15 to 95% of the tumors, respectively. Papillary growth was present in 0 to 20% of the cases (Fig. 2E). Only one case showed a predominantly solid/trabecular growth (Fig. 4B). All cases showed subtle nuclear features with respect to nuclear elongation and chromatin alterations (Figs. 1B and E, 3C, 4C, 5B and D) compared to those identified in most “*BRAF*-like” PTCs. Other well-developed nuclear features, including nuclear groove and pseudoinclusion, were only focally found in five cases (Figs. 1B and E, 5B) and were frequent in the remaining three cases (Figs. 3C, 4C, and 5D). Overall, five cases (62.5%) showed only focal areas present with well-developed PTC nuclear features (nuclear score of ≥ 2). The rest of three cases (38.5%) showed relatively discernable nuclear grooves and pseudoinclusions. In contrast, psammoma bodies, which are typically enriched in “*BRAF*-like” PTCs, were present in five cases. Necrosis or increased mitotic count was absent. Lymphatic invasion was noted in four cases and vascular invasion (angioinvasion) was present in two cases. Coexisted incidental subcentimeter PTCs (size: 0.1–0.2 cm) had been found in four cases. The BRAF p.V600E mutation–specific VE1 IHC was performed on four incidental subcentimeter PTCs. Three of them were positive for VE1 IHC. The one tumor negative for VE1 IHC was also negative for ALK IHC. For the background thyroid parenchyma, three cases (cases 5, 6, and 7) had underlying chronic lymphocytic thyroiditis and one case (case 4) showed follicular nodular disease.

**Table 3:** The pathologic features of *ALK*-rearranged papillary thyroid carcinoma (PTC)

Case No.	Final pathologic diagnosis	Growth pattern (%)				Nuclear features				Tumor border	Other finding				
		Macrofollicular	Microfollicular	Papillary	Solid/trabecular	Elongation	Chromatin alterations	Groove	Pseudoinclusion		Psammoma body	ETE	LI	VI	Non-tumor
1	STPTC	< 5	45	< 5	50	Reduced	Reduced	Frequent	Frequent	Multinodular permeative	+	-	+	+	-
2	IFVPTC	50	45	< 5	0	Reduced	Reduced	Focal	Focal	Multinodular permeative	-	-	+	-	-
3	IFVPTC	50	45	< 5	0	Reduced	Reduced	Focal	Focal	Multinodular permeative	+	+	-	-	-
4	CPTC with follicular growth	45	35	20	0	Reduced	Reduced	Focal	Focal	Multinodular permeative	-	-	-	-	FND
5	CPTC with follicular growth	80	15	5	0	Reduced	Reduced	Focal	Focal	Well-circumscribed	-	-	-	-	-
6	CPTC with follicular growth	30	60	10	0	Reduced	Reduced	Focal	Focal	Multinodular permeative	+	-	-	-	LT
7	IFVPTC	5	95	0	0	Reduced	Reduced	Frequent	Frequent	Multinodular permeative	+	-	+	-	LT
8	IFVPTC	< 5	90	< 5	5	Reduced	Reduced	Frequent	Frequent	Multinodular permeative	+	-	+	+	LT

*STPTC* solid/trabecular subtype PTC, *IFVPTC* infiltrative follicular variant PTC, *CPTC* classic subtype PTC, *ETE* extrathyroidal extension, *LI* lymphatic invasion, *VI* vascular invasion, *FND follicular nodular disease*, *LT* lymphocytic thyroiditis

## Discussion

In this study, we examined the cytologic characteristics, the distribution of the TBS categories, and the histologic correlates of a series of eight *ALK*-rearranged PTCs. In our series, the original FNA cytology diagnosis yielded a notable false-negative result (TBS II) in three out of eight patients. Following the review of seven cases with available FNA slides, we excluded one TBS II case with missed sampling for the final analysis. Two cases initially designated as TBS II were reclassified to TBS III due to the presence of scant microfollicles. Another case originally classified as TBS III remained the diagnosis of TBS III after review based on rare nuclear grooves and pseudoinclusions. The high rate of TBS III interpretation (50%, 3/6) in our series likely resulted from subtle nuclear features and the presence of only microfollicular pattern. Two cases (33.3%, 2/6) were diagnosed as TBS V based on reduced nuclear elongation and chromatin alterations as well as focal grooves and pseudoinclusions. Only one case (16.7%, 1/6) met the criteria for TBS VI. In a case report, Jurkiewicz et al. illustrated a *STRN::ALK* PTC with preoperative FNA diagnosis of TBS III, displaying focal cytological atypia with mild nuclear enlargement and occasional nuclear grooves [13]. Panebianco et al. also observed attenuated nuclear features in FNA cytology of *ALK*-rearranged PTC and a high rate of indeterminate diagnoses (TBS III: 66%, TBS IV: 10%, TBS V: 16%) [12]. The corresponding pathological features of *ALK*-rearranged PTCs in this study showed predominant follicular growth and rare papillary growth in a subset of cases. Limited nuclear elongation and reduced chromatin alterations were also observed. Nuclear grooves and pseudoinclusions were infrequently identified and were obvious in only three of eight cases (37.5%). The findings between cytology and pathology overlapped with good correlation in our study.

The terms “*BRAF*-like” and “*RAS*-like” was first applied in The Cancer Genome Atlas (TCGA) study to describe gene expression pattern in PTCs [2]. TCGA study identified that *ALK*-rearranged PTCs had a neutral gene expression profile which was intermediate between those of *BRAF* p.V600E mutant tumors (“*BRAF*-like”) and *RAS* mutant tumors (“*RAS*-like”) based on their *BRAF-RAS* scoring system. Recently, a few studies and expert reviews have adopted “*BRAF*-like” and “*RAS*-like” designations to describe the cytomorphologic features that are most commonly associated with tumors with the *BRAF* p.V600E mutation (or tumors with a similar gene expression profile) as “*BRAF*-like” whereas tumors with a *RAS* mutation (or tumors with a similar gene expression profile to tumors with a *RAS* mutation) show “*RAS*-like” nuclear atypia [16–18]. While “*BRAF*-like” nuclear atypia is characterized by classical chromatin margination (nuclear clearing) and heterogenous nuclear membrane irregularities resulting in intranuclear pseudoinclusions, “*RAS*-like” tumors are characterized by attenuated chromatin margination and reduced nuclear irregularity. The data from our study showed that *ALK*-rearranged tumors usually demonstrate “*RAS*-like” nuclear atypia rather than “*BRAF*-like” atypia. Interestingly, Chou et al. also described that the majority of *ALK*-rearranged PTCs (eight out of 14 cases) showed a predominance of follicular growth and variable nuclear atypia, which would be considered morphologically “*RAS*-like” [8].

To date, approximately 100 cases of *ALK*-rearranged PTCs have been reported. The clinicopathologic features of *ALK*-rearranged PTCs are summarized in Table 4. *ALK*-rearranged PTCs constitute an overall 2.4% of PTCs, with a female predominance (female to male ratio of 3.56) and a younger age at onset compared to non-*ALK*-rearranged PTC patients [7–11, 19–32]. The majority of *ALK*-rearranged PTCs were PTCs with a mixed papillary and follicular growth (53%), followed by classic (22.9%), solid/trabecular (9.7%), diffuse sclerosing (9.7%), tall cell (2.4%), oncocytic (1.2%), and Warthin-like (1.2%) subtypes [7–13, 19–34]. Our findings aligned with the descriptions in previous literature of a predominance of female patients and various histological subtypes/variants of *ALK*-rearranged PTCs.

**Table 4:** Summary of previous studies of *ALK*-rearranged papillary thyroid carcinoma (PTC)

Reference	Total PTC case number	ALK-rearranged PTC (%)	Female: male	Average age of ALK-rearranged PTC (range)	Pathologic subtypes and features	ALK fusions	Follow-up status
Non-radiation-exposed patients (general population, mainly adults)							
[8]	498	11 (2.2)	11:0	38 (13–68)	6 FVPTC 2 PTC with follicular growth 1 TCPTC 1 OCPTC 1 WLPTC	8 *EML4::ALK* 6 unknown	NED (67.3 months)
	23^a^	3 (13)	3:0	n/a	3 DSPTC		n/a
[32]	43^a^	5 (12)	n/a	n/a	5 DSPTC	1 *EML4::ALK* 2 *STRN::ALK* 2 unknown	n/a
[19]	769	0 (0)	-	-	-	-	-
[7]	307	1 (0.3)	1:0	30	1 FVPTC	1 unknown	NED (63 months)
[20]	126^b^	2 (1.6)	n/a	n/a	2 FVPTC	1 *EML4::ALK* 1 *TFG::ALK*	NED in one case
[21]	16^c^	1 (6.3)	n/a	n/a	1 subcentimeter PTC with S/T growth	1 *STRN::ALK*	NED
[22]	59^d^	1 (1.7)	0:1	60	1 CPTC	1 *EML4::ALK*	AWD (60 months)
[23]	116^e^	4 (3.5)	3:1	42 (34–47)	2 CPTC 2 FVPTC	4 *STRN::ALK*	NED
[24]	29^e^	2 (6.9)	n/a	n/a	2 CPTC	2 *STRN::ALK*	n/a
[25]	79^f^	0 (0)	-	-	-	-	-
	262^g^	4 (1.5)	3:1	37 (13–50)	2 CPTC 1 FVPTC 1 STPTC	2 *STRN::ALK* 2 unknown	NED (26.2 months)
[26]	235	1 (0.4)	n/a	n/a	1 PTC with follicular growth	1 *STRN::ALK*	n/a
	21^h^	3 (14.3)	n/a	n/a	3 PTC with follicular growth	2 *STRN::ALK* 1 *EML4::ALK*	n/a
[27]	14^h^	1 (7.1)	0:1	27	1 CPTC	1 *EML4::ALK*	n/a
[12]	-	27	22:5	38 (8–69)	9 CPTC 8 FVPTC 10 PTC with follicular growth	13 *STRN::ALK* 11 *EML4::ALK* 2 *CTSB::ALK* 1* PPP1R21::ALK*	NED in 10 cases (55.9 months)
[34]	-	2	1:1	49 (32, 66)	2 PTC with follicular growth	1 *STRN::ALK* 1 *EML4::ALK*	NED (19 and 96 months)
[13]	-	1	1:0	44	1 CPTC	1 *STRN::ALK*	n/a
[33]	-	1	0:1	62	1 TCPTC	1 *EML4::ALK*	Recurrence
Non-radiation-exposed pediatric and young adult patients							
[28]	41^i^	3 (7.3)	3:0	10 (7–15)	3 PTC, not specified subtype	1 *STRN::ALK* 1 *EML4::ALK* 1 *GTF2IRD1::ALK*	n/a
[29]	51^j^	1 (2)	0:1	< 10	1 FPTC	1 *EML4::ALK*	n/a
[10]	93^k^	6 (6.5)	4:2	14 (9–20)	1 CPTC 4 FVPTC 1 PTC with follicular growth	6 *STRN::ALK*	n/a
Radiation-exposed patients							
[11]	79^l^	10 (12.7)	n/a	33 in PTC with S/T growth 53 in PTC, not specified subtype	6 PTC with S/T growth 4 PTC, not specified subtype	6 *EML4::ALK* 4 unknown	n/a
[9]	63^m^	1 (1.6)	n/a	n/a	1 PTC, not specified subtype	1 *STRN::ALK*	n/a
[30]	65^n^	5 (7.7)	n/a	23.2	5 PTC, not specified subtype	5 *STRN::ALK*	n/a
[31]	77^n^	7 (9.1)	5:2	23.1	7 PTC, not specified subtype	2 *EML4::ALK* 5 unknown	n/a

*CPTC* classic subtype PTC, *FVPTC* follicular variant PTC, TCPTC tall cell subtype PTC, *OCPTC* oncocytic subtype PTC, *WLPTC* Warthin-like subtype PTC, *DSPTC* diffuse sclerosing subtype PTC, *STPTC* solid/trabecular subtype PTC, *S/T* solid/trabecular, *NED* no evidence of disease, *AWD* alive with disease

^a^Only cases of DSPTC

^b^126 cases of FVPTC with tumor size more than 1 cm

^c^16 cases of subcentimeter PTC with lymph node metastasis

^d^59 cases of radioactive iodine-refractory PTC

^e^Study using RT-PCR aimed for *STRN::ALK* fusion

^f^79 cases of BRAF-mutant PTC

^g^262 cases of BRAF-negative PTC

^h^PTC negative for mutations of *BRAF*, *HRAS*, *NRAS*, and *KRAS* as well as rearrangements of *RET*, and *PAX8::PPARG*

^i^41 cases of PTC with patients’ age under 40 years old

^j^51 cases of PTC with patients’ age under 18 years old

^k^93 cases of PTC with patients’ age between 6 and 20 years old

^l^79 cases of PTC in atomic bomb survivors

^m^63 cases of PTC with patients’ age under 18 years old with radiation exposure after Fukushima accident

^n^Patients with radiation exposure after Chernobyl accident

The majority of *ALK*-rearranged thyroid carcinomas were PTCs (87.3%), followed by poorly differentiated thyroid carcinomas (11%) and anaplastic thyroid carcinomas (1.7%) [7–13, 20–35]. The most frequent *ALK* fusion variants were *STRN::ALK* (45.8%) and *EML4::ALK* (31.4%). Other less common fusion partner genes included *CTSB*, *GTF2IRD1*, *PPP1R21*, *TFG*, *CCDC149*, and *ITSN2* [12, 20, 28, 36]. Similarly, *STRN::ALK* and *EML4::ALK* fusions comprised the majority of our cases, including three (50%) with *EML4::ALK* and two (33.3%) with *STRN::ALK* fusion among the six cases tested. We also identified a rare *ITSN2::ALK* fusion gene (case 1), previously reported in only one FNA cytology case [12]. Interestingly, the FNA cytology of *ITSN2::ALK* PTC in our study exhibited the most pronounced PTC nuclear features, consistent with the diagnostic criteria of TBS VI (Fig. 4). The histological examination revealed more frequent PTC nuclear features than *EML4::ALK* and *STRN::ALK* fusion–associated cases.

The prognosis of *ALK*-rearranged thyroid carcinomas remains uncertain due to its rarity. In our study, all eight cases displayed an absence of high-grade features, and seven showed no signs of recurrence during the follow-up period. Most previously reported PTC cases also exhibited no evidence of disease during the follow-up period [7, 8, 12, 20, 21, 23, 25, 34]. There was only one classic subtype PTC that persisted with disease for 5 years and another tall cell subtype PTC that manifested with recurrence 3 years after thyroidectomy [22, 33]. Although rare, similar to other molecular alterations, *ALK* rearrangement may also occur in aggressive thyroid cancers, such as poorly differentiated or anaplastic thyroid carcinoma [12, 26, 34–37]. Since 2011, several ALK tyrosine kinase inhibitors have been approved for the treatment of non-small cell lung carcinoma [38]. Godbert et al. reported a case of anaplastic thyroid carcinoma showing focal follicular variant PTC component with tumor regression after off-label use of crizotinib [35]. Thus, ALK inhibitors also appear to be a promising therapeutic agent for aggressive thyroid cancer with *ALK* fusions.

## Conclusion

Although *ALK*-rearranged PTCs have been shown to harbor a neutral gene expression profile from a *BRAF-RAS* scoring perspective of TCGA study, one can see tumors with “*RAS*-like” and “*BRAF*-like” nuclear alterations or the combination of both in an individual case. In this current study, the “*RAS*-like” nuclear features were more commonly identified. The diagnostic challenges due to attenuated nuclear alterations and the absence of a discernible papillary architecture seem to result in frequent indeterminate diagnosis of preoperative FNA. The corresponding histopathologic features included a predominant follicular growth along with variable and subtle nuclear features. Recognition of these findings is essential for the identification of *ALK*-rearranged PTC.

## Data Availability

The data that support the findings of this study are available from the corresponding author upon reasonable request.
